# Activation of novel estrogen receptor GPER results in inhibition of cardiocyte apoptosis and cardioprotection

**DOI:** 10.3892/mmr.2015.3674

**Published:** 2015-04-24

**Authors:** WAN-LI LI, WEI XIANG, YE PING

**Affiliations:** 1 Department of Cardiology, Tongji Hospital, Tongji Medical College, Huazhong University of Science and Technology, Wuhan, Hubei 430030, P.R. China; 2 Department of Cardiology, Union Hospital, Tongji Medical College, Huazhong University of Science and Technology, Wuhan, Hubei 430022, P.R. China

**Keywords:** G protein-coupled receptor 30, ischemia-reperfusion injury, cardiocyte apoptosis, cardioprotection

## Abstract

Several studies have recently demonstrated that G protein-coupled estrogen receptor (GPER) 30 directly binds to estrogen and mediates its action. The aim of the present study was to investigate the effects of GPER on cardiocyte apoptosis following ischemia/reperfusion injury (MIRI) in H9C2 myocardial cells. H9C2 cells were treated with a specific GPER agonist (G1), 17β-estradiol (E2) or the vehicle. The cells were subjected to 20 min of myocardial ischemia followed by 120 min of reperfusion. They were then randomly assigned to three experimental groups: Control, G1, E2. B-cell lymphoma 2 (Bcl-2) and Bcl-2 associated X (Bax) levels were measured, Hoechst 33258 staining was performed to assess apoptosis, and superoxide dismutase (SOD), tumor necrosis factor (TNF)-α and adenosine triphosphatase (ATPase) levels were determined. To test the specificity of G1, GPER-knockout cells were treated with G1 and analyzed as stated above. Compared with the vehicle-treated groups, G1 and E2-treated groups exhibited elevated Bcl-2 levels, decreased Bax levels and cell apoptosis, significantly increased SOD and ATP levels and decreased TNF-α levels following ischemia-reperfusion. However, G1 had no evident effects on the GPER-knockout cells. In conclusion, the present study suggested that GPER activation provided a cardioprotective effect following ischemia-reperfusion by inhibiting cardiocyte apoptosis.

## Introduction

Estrogen has protective effects in cardiovascular function (1). The biological effects of estrogen are mainly mediated by estrogen receptors (ERs). Two classic nuclear ER isoforms, ERα and ERβ, are encoded by separate genes and have differential distribution within tissues and cells. These receptors have been demonstrated to be expressed in both neonatal (2) and adult (3) cardiac myocytes.

In addition to the classic ERs, a G protein-coupled ER (GPER) has been found to be expressed in cardiomyocytes (4,5). Notably, the majority of studies which have examined ER localization with cardiac cells did not find a difference in the distribution or abundance between males and females (3,4). Recent studies have demonstrated the existence of a novel G protein-coupled receptor 30, GPR30, here referred to as G protein-coupled estrogen receptor (GPER), that binds directly to estrogen and mediates its action (6–10).

Several studies have discovered coagulable cytolysis around the ischemic myocardial infarction zone without the presence of infiltration of inflammatory cells, a phenomenon similar to the morphological changes of apoptosis (11). Furthermore, apoptotic cells began to appear in the ischemic margin 1 h following ischemia, and the number of the apoptotic cells increased with time and peaked at 5 h following ischemia, suggesting apoptosis may be a main feature of myocardial ischemia (12).

GPER activation improved functional recovery and reduced the infarct size in isolated rat hearts following ischemia and reperfusion (13). The potential role and the mechanism of GPER activation in cardioprotection is an important topic under investigation. The aims of the present study were to examine the role of GPER activation in cardiocyte apoptosis and SOD, TNF-α, ATPase expression by using a specific GPER agonist (G1) (14) in H9C2 myocardial cells following ischemia-reperfusion.

## Materials and methods

### Model preparation

H9C2 myocardial cells were subjected to 20 min of ischemia (in a hypoxia chamber filled with 95% N_2_ and 5% CO_2_ at 37°C) followed by 120 min of reperfusion (with 95% O_2_, 5% CO_2_ at 37°C). The cells were then randomly assigned to three experimental groups (n=5/group): The control G1 (a GPER-specific agonist; 10 nmol/l) and E2 (1 nmol/l) groups (Sigma-Aldrich, St Louis, MO, USA). G1 or 17β-estradiol (E2) was administered 5 min prior to ischemia.

GPER-knockout (KO) H9C2 cells were similarly subjected to 20 min of ischemia and 120 min of reperfusion. They were randomly assigned to five experimental groups (n=5/group); control, empty vector, G1, GPER-KO, GPER-KO+G1.

### Quantitative polymerase chain reaction (qPCR)

Total RNA was extracted from H9C2 cell with the use of TRIzol reagent (Invitrogen Life Technologies, Carlsbad, CA, USA). Isolated RNA (10 *µ*g) was reverse-transcribed using the RevertAid First Strand cDNA Synthesis kit (therm k1622; Thermo Scientific, Waltham, MA, USA). PCR amplification was conducted in a total volume of 25 *µ*l (QPS-201; Toyobo, Osaka, Japan): cDNA 2.5 *µ*l, F (5 pmol/ml) 2 *µ*l, R (5 pmol/ml) 2 *µ*l, THUNDERBIRD™ SYBR Green pPCR Mix 12.5 *µ*l and H_2_O 6 *µ*l, using an Applied Biosystems 7300 Real-Time PCR System (Applied Biosystems Life Technologies, Foster City, CA, USA). The primer sequence for rat GADPH was as follows: F, 5′-CGCTAACATCAAATGGGGTG-3′; and R, 5′-TTGCTGACAATCTTGAGGGAG-3′. GAPDH was used as an internal control. Cycling conditions were: 95°C for 1 min (95°C for 15 sec, 58°C for 20 sec, 72°C for 20 sec) for 40 cycles, and 82°C for 10 min. Data were calculated using the 2^−ΔΔCt^ method.

### Western blot analysis

Protein (40 *µ*g) from H9C2 cells was separated oby SDS-PAGE and electrophoretically transferred onto polyvinylidene fluoride membranes. The membranes were incubated with Bcl-2 (rabbit monoclonal; Cell Signaling Technology, Inc., Danvers, MA, USA) and Bax (rabbit monoclonal; Abcam, Cambridge, UK) antibodies. The blots were then washed and incubated with horseradish peroxidase-conjugated secondary antibody (KPL, Inc., Gaithersburg, MD, USA) and the blot was developed with a supersignal enhanced chemiluminescence detection kit (Thermo Scientific). The blots were scanned using an Epson V300 scanner (Epson, Suwa, Japan) and the blot densities were analyzed with AlphaEaseFC software (Alphalnnotech, San Leandro, CA, USA).

### Identification of apoptotic cells with Hoechst staining

Apoptotic cells were determined using Hoechst 33258 staining. The cells were washed in phosphate-buffered saline and labeled with Hoechst 33258 (Invitrogen Life Technologies) at room temperature in the dark for 10 min. The cell nuclei were observed and visualized by an inverted fluorescence microscope (Nikon-Ti; Nikon Corporation, Tokyo, Japan). The number of apoptotic nuclei was determined in at least six randomly selected areas from three coverslips of every experimental group. The data were expressed as the percentage of apoptotic cells relative to the total number of cells.

### SOD

Measurement of SOD enzyme activation was assessed with xanthine oxidase enzyme kit (Nanjing Jiancheng Bioengineering Institute, Nanjing, China) following the manufacturer’s instructions and measured with an autoanalyzer (752-P UV-visible spectrophotometer; Xianguang Instrument Co., Ltd. Shanghai, China) at 550 nm and 37°C.

### TNF-α

TNF-α was measured using commercially available quantitative sandwich ELISA kits according to manufacturer’s instructions. The analyses were performed with 96-well microtiter plate ELISA kits for TNF-α (eBioscience, San Diego, CA, USA). Microtiter strips pre-coated with biotinylation antibodies generated against the proteins were used for quantification.

### ATPase

Measurement of ATPase activation was measured via inorganic phosphorus activation. The samples were analyzed according to the manufacturer’s instructions (Nanjing Jiancheng Bioengineering Institute) and measured with the 752-P UV-visible spectrophotometer at 636 nm and room temperature.

### Statistical analysis

All of the data were subjected to analysis of variance, followed by a Bonferroni correction for post-hoc t-test using the SAS version 9.3 software (SAS Institute Inc., Cary, NC, USA). P≤0.05 was considered statistically significant.

## Results

### Pretreatment with G1 and E2 increases Bcl-2 mRNA and protein expression in H9C2 cells following I/R

**Figure 1:**
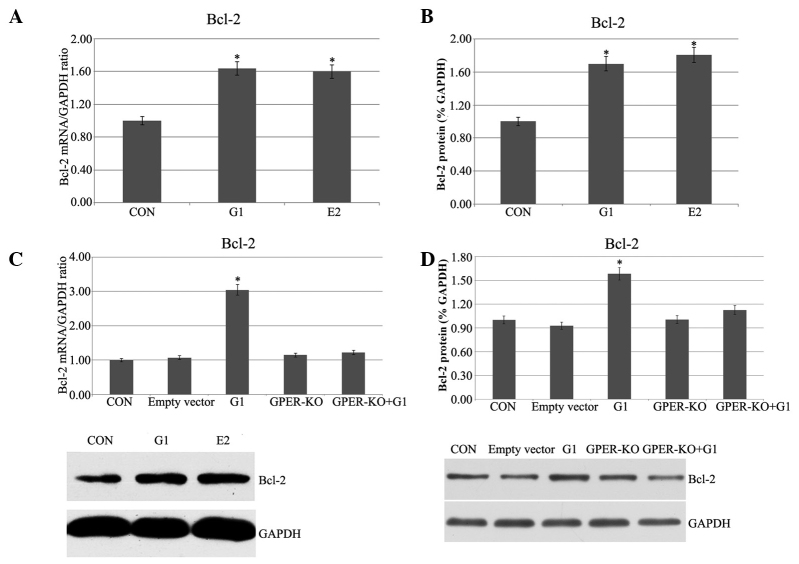
(A) Myocardial Bcl-2 mRNA expression. CON, H9C2 cells were subjected to 20 min of ischemia followed by 120 min of reperfusion; G1, G1 (10 nmol/l) was administered 5 min prior to ischemia; E2, E2 (1 nmol/l) was administered5 min prior to ischemia. (B) Myocardial Bcl-2 protein expression. (C and D) Bcl-2 mRNA and protein expression, respectively, in GPER-KO H9C2 cells. (P<0.05 vs. control). GPER, G protein-coupled estrogen receptor; E2, 17β-estradiol; G1, GPER agonist; CON, control; KO, knockout; Bcl-2, B-cell lymphoma 2.

In the G1 and E2 groups, in which the H9C2 cells had been subjected to 20 min of ischemia followed by 120 min of reperfusion, the Bcl-2 mRNA expression, as measured by qPCR, was higher than that in the control group (Fig. 1A). To further confirm the relative expression of Bcl-2 in both strains, western blot analysis was also performed, and the Bcl-2 protein expression was significantly enhanced in the G1 and E2 groups (Fig. 1B). To confirm that the G1-induced effects were due to the specific activation of GPER, G1 was administered 5 min prior to ischemia in the GPER-knockout H9C2 cells. No evident effects were found in the GPER-knockout H9C2 cells (Fig. 1C and D). Therefore, it is evident that Bcl-2 mRNA and protein expression were increased in the I/R myocardial cells by G1 or E2, and the G1-induced activation was dependent on activation of GPER.

### Pretreatment with G1 and E2 decreases Bax mRNA and protein expression in H9C2 cells following I/R

**Figure 2:**
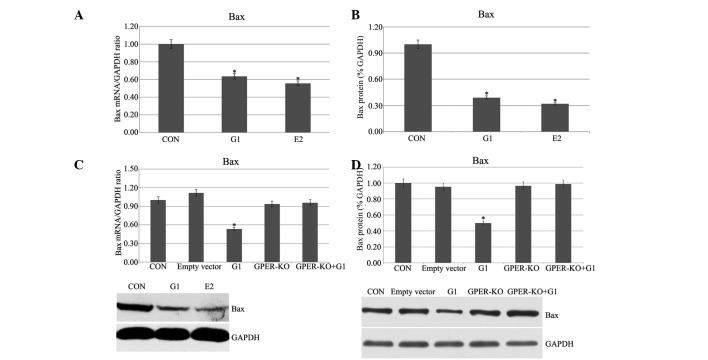
(A and B) Bax mRNA and protein expression, respectively, in I/R H9C2 cells. (C and D) Bax mRNA and protein expression, respectively, in GPER-knockout H9C2 cells. (^*^P<0.05 vs. control). I/R, ischemia/reperfusion; GPER, G protein-coupled estrogen receptor; E2, 17β-estradiol; G1, GPER agonist; CON, control; KO, knockout; Bax, B-cell lymphoma-associated X.

Following administration of G1 or E2, a marked decrement of Bax expression was observed as determined by qPCR and western blotting experiments (Fig. 2A and B). G1 had no evident effects on the GPER-knockout H9C2 cells (Fig. 2C and D). The observed stable Bax expression suggested that G1 and E2 inhibited Bax in I/R H9C2 cells, and the G1-induced activation was through the activation of GPER.

### G1 and E2 protect H9C2 myocardial cells from I/R-induced apoptosis

**Figure 3:**
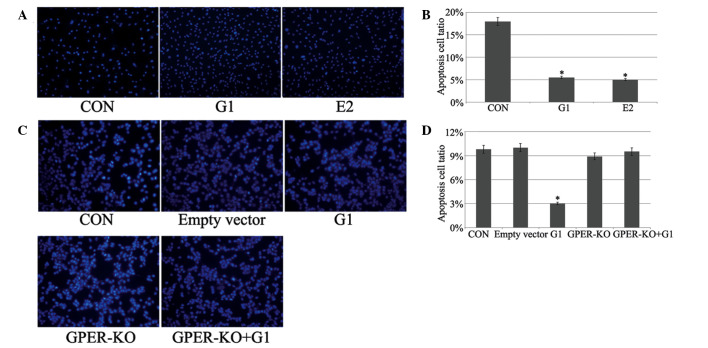
Effect of G1 or E2 on apoptosis in H9C2 cells. Apoptotic cells were detected by Hoechst 33258 staining. (A and C) Representative fluorescence images of nuclear staining with Hoechst 33258. A, magnification x100; C, magnification x200. Smaller, brighter cells were considered to be apoptotic. (B and D) Quantitative analysis of apoptotic nuclei in cultures. Data are expressed as the mean ± standard error of the mean (^*^P<0.05 vs. control). GPER, G protein-coupled estrogen receptor; E2, 17β-estradiol; G1, GPER agonist; CON, control; KO, knockout.

Apoptotic cells were determined by Hoechst 33258 staining, which allows determination and quantification of cells with fragmented and condensed chromatin. Fig. 3 demonstrates that G1 or E2 treatment decreased apoptosis in H9C2 cells. No evident changes were observed in the GPER-knockout H9C2 cells.

### Pretreatment with G1 and E2 increases SOD levels in H9C2 cells following I/R

**Figure 4:**
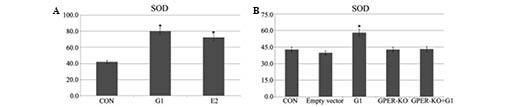
(A) SOD following 120 min of reperfusion was significantly higher in the G1 and E2 groups vs. the control. (B) No evident effects of G1 were found in the GPER-knockout H9C2 cells. (^*^P<0.05 vs. control). SOD, superoxide dismutase; GPER, G protein-coupled estrogen receptor; E2, 17β-estradiol; G1, GPER agonist; CON, control; KO, knockout.

Application of G1 or E2 5 min prior to ischemia induced an increase in SOD levels in the H9C2 cells subjected to 20 min of ischemia followed by 120 min of reperfusion (Fig. 4A). In the GPER-knockout H9C2 cells, the increase was not evident (Fig. 4B).

### G1 and E2 decrease levels of TNF-α following I/R

**Figure 5:**
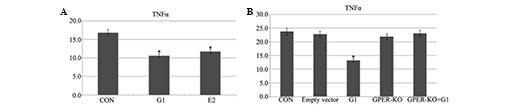
(A) TNF-α following 120 min of reperfusion was significantly lower in the G1 and E2 groups vs. the control. (B) No evident changes in GPER-knockout H9C2 cells were observed. (^*^P<0.05 vs. control). TNF-α, tumor necrosis factor-α; GPER, G protein-coupled estrogen receptor; E2, 17β-estradiol; G1, GPER agonist; CON, control; KO, knockout.

The ELISA demonstrated that administration of G1 or E2 prior to ischemia-reperfusion decreased the levels of TNF-α as compared with the control group (Fig. 5A). There were no further effects in the GPER-knockout H9C2 cells (Fig. 5B).

### G1 and E2 increase levels of ATPase following I/R

**Figure 6:**
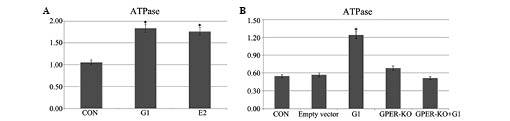
(A) ATPase following 120 min of reperfusion was significantly higher in the G1 and E2 groups vs. the control. (B) There were no further effects in GPER-knockout H9C2 cells. (^*^P<0.05 vs. control). ATPase, adenosine triphosphatase; GPER, G protein-coupled estrogen receptor; E2, 17β-estradiol; G1, GPER agonist; CON, control; KO, knockout.

The administration of G1 or E2 in H9C2 cells induced elevated ATPase levels following 20 min of ischemia and 120 min of reperfusion (Fig. 6A). In the GPER-knockout H9C2 cells, this elevation was no evident (Fig. 6B).

## Discussion

The present study reported that the activation of GPER by the specific agonist G1 protects the heart against I/R injury, inhibiting cardiocyte apoptosis.

GPER has been demonstrated to be localized in the endoplasmic reticulum (8,15) and plasma membrane (16) of reproductive organs, uterus and mammary glands, as well as in hippocampal regions (17). The results of the present study demonstrated the protection of H9C2 cells via activation of GPER. Haas *et al* (18) concluded that GPER contributes to the regulation of blood pressure and vascular tone, suggesting the possibility that a number of the known vasculoprotective effects of estrogen involve GPER activation. Jean and Mansoureh (19) reported the acute G1 treatment significantly reduced the infarct size following ischemia-reperfusion in isolated hearts from male mice. In the present study, it was identified that GPER activation inhibited cardiocyte apoptosis, significantly increased SOD, ATP and decreased the TNF-α expression level following ischemia-reperfusion. Additionally, the G1-mediated effects were eradicated in H9C2 cells. Taken together, these results provide evidence that GPER is the primary receptor responsible for long-term cellular changes leading to cardioprotection in H9C2 cells. Therefore the cardioprotective action of estrogen may involve the activation of GPER.

Besides activation by E2, each of these ER isoforms has specific pharmacological agonists. Bopassa *et al* (19) have demonstrated that acute G1 treatment considerably improves the recovery of cardiac function following ischemia-reperfusion. Similarly, a recent study demonstrated that G1 treatment of isolated rat hearts is cardioprotective during ischemia-reperfusion (13).

Clinically, myocardial ischemia-reperfusion is an acute and severe injury and the extensive apoptosis and necrosis of cardiomyocytes at the earliest stage of reperfusion accounts for the majority of clinical manifestations (20). For a long time, necrosis was regarded as the sole cause of cell death in myocardial ischemia-reperfusion injury. However, a recent study indicated that apoptosis also has an important role in the process of cardiomyocyte damage (21). Bcl-2 and Bax are two proteins that are directly involved in apoptosis signaling. Therefore, in the present study, the contribution of Bcl-2 and Bax to myocardial cell apoptosis induced by I/R was examined. The mRNA and protein levels of Bcl-2 were evaluated 120 min following reperfusion subsequent to pretreatment with E2 or G1; however, the mRNA and protein expression of Bax decreased 120 min following reperfusion. The above cells were stained with Hoechst 33258 for detecting apoptotic cells. Compared with the cells subjected to I/R, pretreatment with E2 and G1 inhibited cell apoptosis. To confirm that the G1-induced effects were due to the specific activation of GPER, G1 was administered 5 min prior to ischemia in GPER-knockout H9C2 cells. No evident effects were found in the GPER-knockout H9C2 cells.

It is well established that the activation of SOD during I/R has an important role in the modulation of the cardioprotection. Liesa *et al* (22) reported that increasing SOD shortly prior to the onset of ischemia represents an improved strategy to improve the functional recovery from I/R. The present study demonstrated an increase in SOD levels induced by administering G1 and E2 in H9C2 cells prior to ischemia-reperfusion. In the GPER-knockout H9C2 cells, no elevation was identified. These data indicated that GPER activation by G1 induced cardioprotection.

Despite the appearance of numerous cytokines following myocardial ischemia, the elevation of TNF-α expression was shown to only be apparent during reperfusion (23,24). TNF-α may have an important role during the activation of the neutrophilic granulocytes (12). Pro-inflammatory cytokines, including TNF-α, have emerged as significant contributors to myocardial dysfunction (25). In the present study, it was demonstrated that E2 and G1 treatment lead to the degradation of TNF-α following I/R. By contrast, G1 had no evident effects on the GPER-knockout H9C2 cells.

It was also identified that ATPase expression was enhanced in E2- and G1-pretreated H9C2 cells following I/R, and this effect of G1 was eliminated in GPER-knockout H9C2 cells.

In conclusion, the present study demonstrated that GPER activation protected cadiocytes following ischemia-reperfusion, which was evident from the inhibition of apoptosis, elevation of SOD and ATPase as well as reduction of TNF-α. These results enhance the understanding of the GPER activation-induced cardioprotection following ischemia-reperfusion. Additionally, the present study offers a potentially unique therapy in terms of a selective estrogen receptor modulator that confers cardio-protection without the increased risk for cancer. However, the mechanism through which the activation of GPER inhibits cardiocyte apoptosis remains to be investigated.
